# New Chimeric Porcine Coronavirus in Swine Feces, Germany, 2012

**DOI:** 10.3201/eid2207.160179

**Published:** 2016-07

**Authors:** Valerij Akimkin, Martin Beer, Sandra Blome, Dennis Hanke, Dirk Höper, Maria Jenckel, Anne Pohlmann

**Affiliations:** Chemical and Veterinary Investigations Office Stuttgart, Fellbach, Germany (V. Akimkin);; Friedrich-Loeffler-Institut, Greifswald–Insel Riems, Germany (M. Beer, S. Blome, D. Hanke, D. Höper, M. Jenckel, A. Pohlmann)

**Keywords:** porcine coronavirus, coronavirus, viruses, chimeric virus, porcine epidemic diarrhea virus, PEDV, transmissible gastroenteritis virus, TGEV, recombination, pigs, feces, Germany

**To the Editor:** Porcine epidemic diarrhea virus (PEDV) and transmissible gastroenteritis virus (TGEV) can cause severe enteritis in pigs accompanied by diarrhea, vomiting, and dehydration. Clinical signs are most prominent in young suckling pigs, in which high mortality rates are common. As seen in recent porcine epidemic diarrhea outbreaks in the United States and Asia, the effect on the pig industry can be tremendous.

Recently, Boniotti et al. (*1*) reported detection and genetic characterization of swine enteric coronaviruses (CoVs) circulating in Italy during 2007–2014. Characterization was based on sequencing and phylogenetic analyses of spike genes of TGEV and PEDV isolates. This study also reported a new recombinant CoV strain with a TGEV backbone and a PEDV spike gene (SeCoV/Italy/213306/2009; KR061459), which was identified as a swine enteric CoV (SeCoV). This chimeric virus presumably resulted from a recombination event.

Accompanying a study of recent porcine epidemic diarrhea cases in Germany caused by a new PEDV Indel strain (*2*), we retrospectively analyzed fecal samples from pigs that showed typical clinical symptoms of a PEDV infection. The sample set included fecal material collected from a farm in southern Germany on which an episode of diarrhea among pigs occurred in 2012. This material was shown by electron microscopy to contain CoV-like particles (Figure), but showed negative results by reverse transcription PCRs specific for the PEDV nucleocapsid gene.

**Figure:**
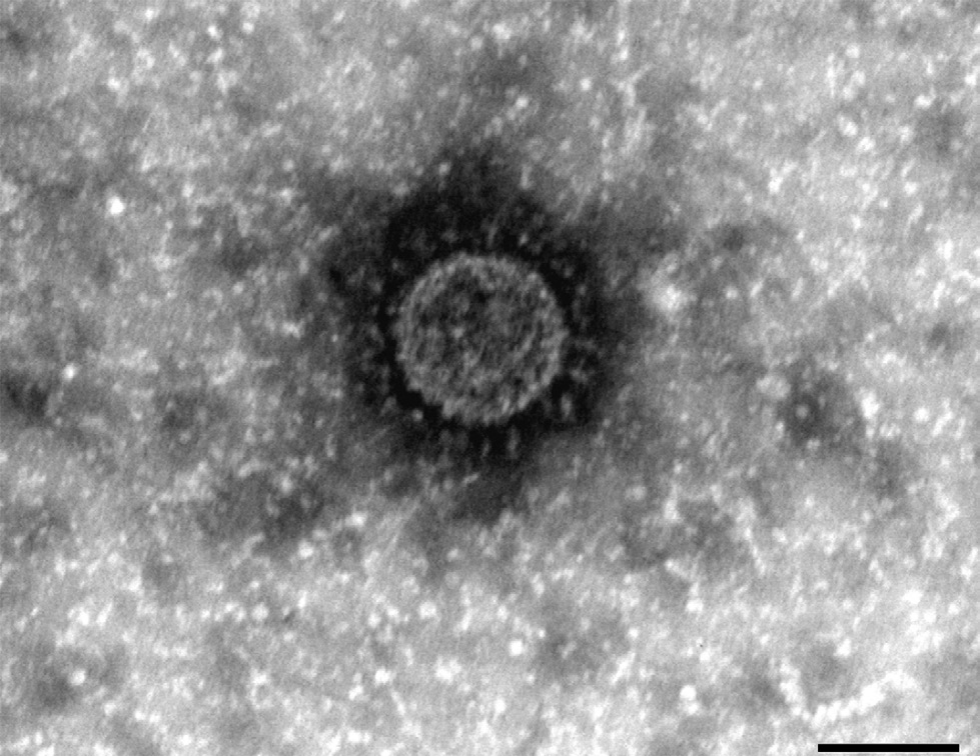
Electron micrograph of a new chimeric swine enteric coronavirus (SeCoV/GER/L00930/2012), Germany, 2012. Scale bar indicates 100 nm.

Subsequent metagenomic analyses resulted in the full-genome sequence of a swine enteric CoV (SeCoV/GER/L00930/2012). We found a sequence showing high similarity (99.5% identity) with the TGEV/PEDV recombinant reported by Boniotti et al. (*1*). Network analysis of complete genome sequences of similar CoVs underline the chimeric nature of the genome between TGEV and PEDV genome sequences (Technical Appendix Figure, panel A). The chimeric nature of the virus strain was confirmed by RT-PCR with primers spanning possible recombination sites and analysis of overlapping reads from next-generation sequencing.

Annotation of the sequence of SeCoV/GER/L00930/2012 performed on the basis of SeCoV/Italy/213306/2009 identified a similar putative coding sequence with a TGEV backbone and a spike coding sequence similar to that for PEDV (Technical Appendix panel B). Downstream of the spike protein–coding open reading frame (ORF), an additional hypothetical ORF was identified in both SeCoV sequences. The coded amino acid sequences (27 aa in the virus from Germany and 30 aa in the virus from Italy) resembled an N- and C-terminally truncated TGEV nonstructural protein 3a. The difference of 3 aa between the 2 strains is the result of a 10-bp deletion at the 3′-end of the hypothetical ORF, which shifted the stop 3 codons to the 5′- end (Technical Appendix Figure, panel B) in SeCoV/GER/L00930/2012. This deletion is apparently located within the potential 3′ recombination site (Technical Appendix Figure, panel B).

It is tempting to speculate that SeCoV/Italy/213306/2009 is a precursor of SeCoV/GER/L00930/2012, and that other members of this novel genotype are still undetected. These viruses might be targets of secondary mutation and recombination events. Therefore, more chimeric CoVs should be identified to determine the potential origin of the recombination event.

In conclusion, we detected an enteric CoV that resembled the TGEV/PEDV chimeric virus reported by Boniotti et al. (*1*). Although these findings support the notion that CoV genomes are subject to mutations and recombination events, problems in disease diagnosis can be foreseen. In countries where porcine epidemic diarrhea, transmissible gastroenteritis, or both of these diseases are reportable, correct diagnosis and reporting might be difficult. Thus, diagnosticians should be aware of possible recombinants of swine CoVs. Diagnostic problems can be prevented by use of a double-check strategy with techniques specific for different genome regions. Apart from diagnostic obstacles, the effect of virus recombinations in terms of virulence and organ tropism is unknown and needs further investigations.

**Technical Appendix.** Analysis of a new chimeric swine enteric coronavirus (SeCoV/GER/L00930/2012), Germany, 2012.
