# Supplementary material for: New Chimeric Porcine Coronavirus in Swine Feces, Germany, 2012
Source: Emerg Infect Dis. 2016 Jul;22(7):1314–5. doi: 10.3201/eid2207.160179 (PMC4918154; doi:10.3201/eid2207.160179)
Supplement: Supplementary file 1 — Technical Appendix. Analysis of a new chimeric swine enteric coronavirus (SeCoV/GER/L00930/2012), Germany, 2012. [file 16-0179-Techapp-s1.pdf]

Article DOI: http://dx.doi.org/10.3201/eid2207.160179

# New Chimeric Porcine Coronavirus in Swine Feces, Germany, 2012

## Technical Appendix

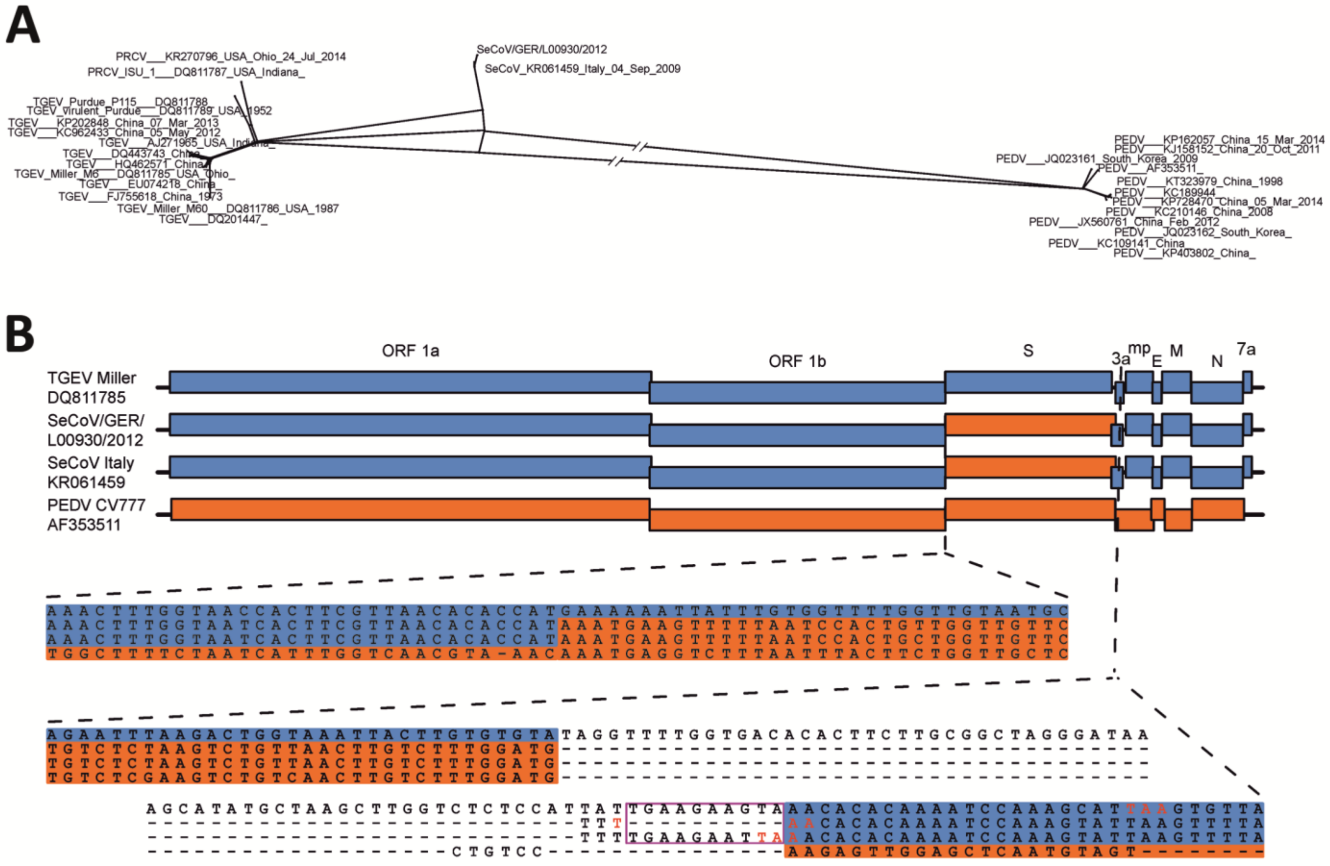


**Technical Appendix Figure.** Analysis of a new chimeric swine enteric coronavirus (SeCoV/GER/L00930/2012), Germany, 2012. A) Network analysis of complete genomes of SeCoVs and related coronaviruses. PRCV, porcine respiratory coronavirus; PEDV, porcine epidemic diarrhea virus; TGEV, transmissible gastroenteritis virus. B) Top: schematic representation (not to scale) of 2 chimeric viruses and their putative progenitors. Sequences most related to progenitor strain TGEV Miller are indicated in blue, and sequences most related to PEDV CV777 are indicated in red. Protein designations are according to the International Committee on Taxonomy of Viruses. ORF, open reading frame; S, spike; 3a, accessory proteins encoded by ORF3; mp, alphacoronavirus-specific accessory membrane protein amp; E, envelope; M, membrane; N, nucleocapsid; 7a, accessory proteins encoded by ORF7. Bottom: alignments of the putative recombination sites. A deletion in SeCoV from Germany is indicated by the purple box, and stop codons of the hypothetical nonstructural protein 3a coding ORFs are indicated in red.
